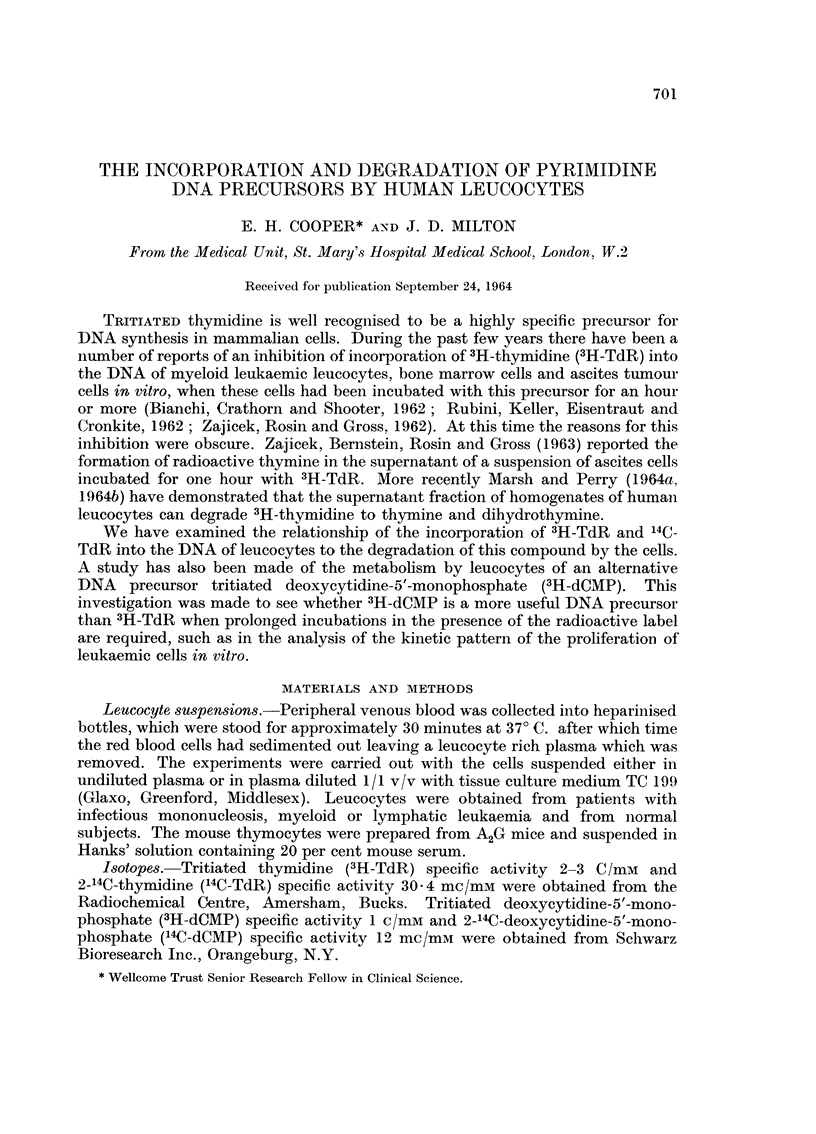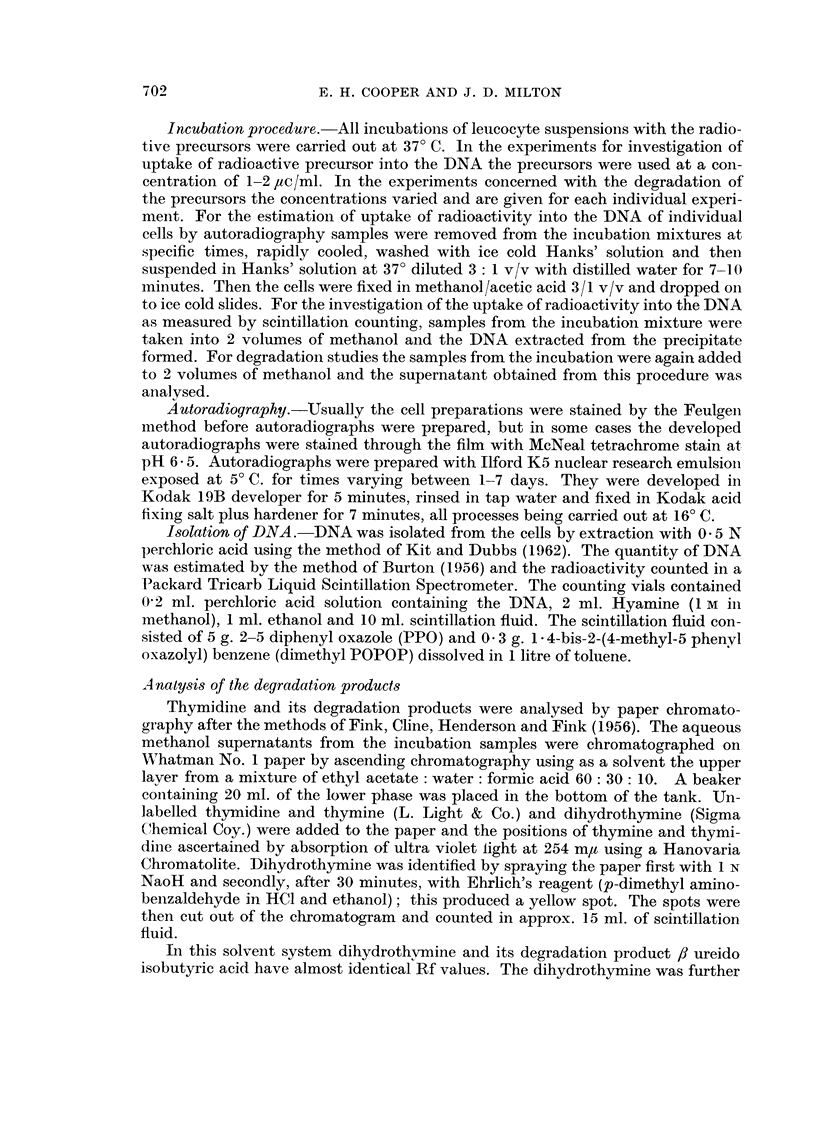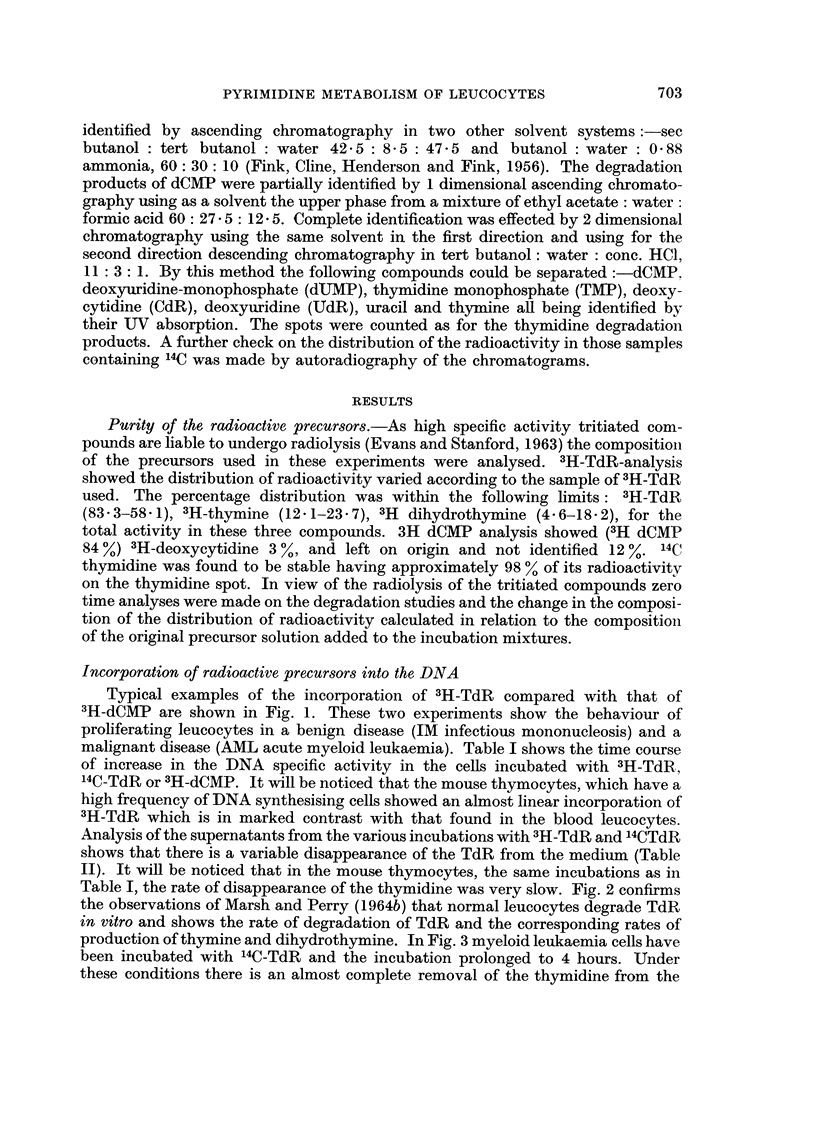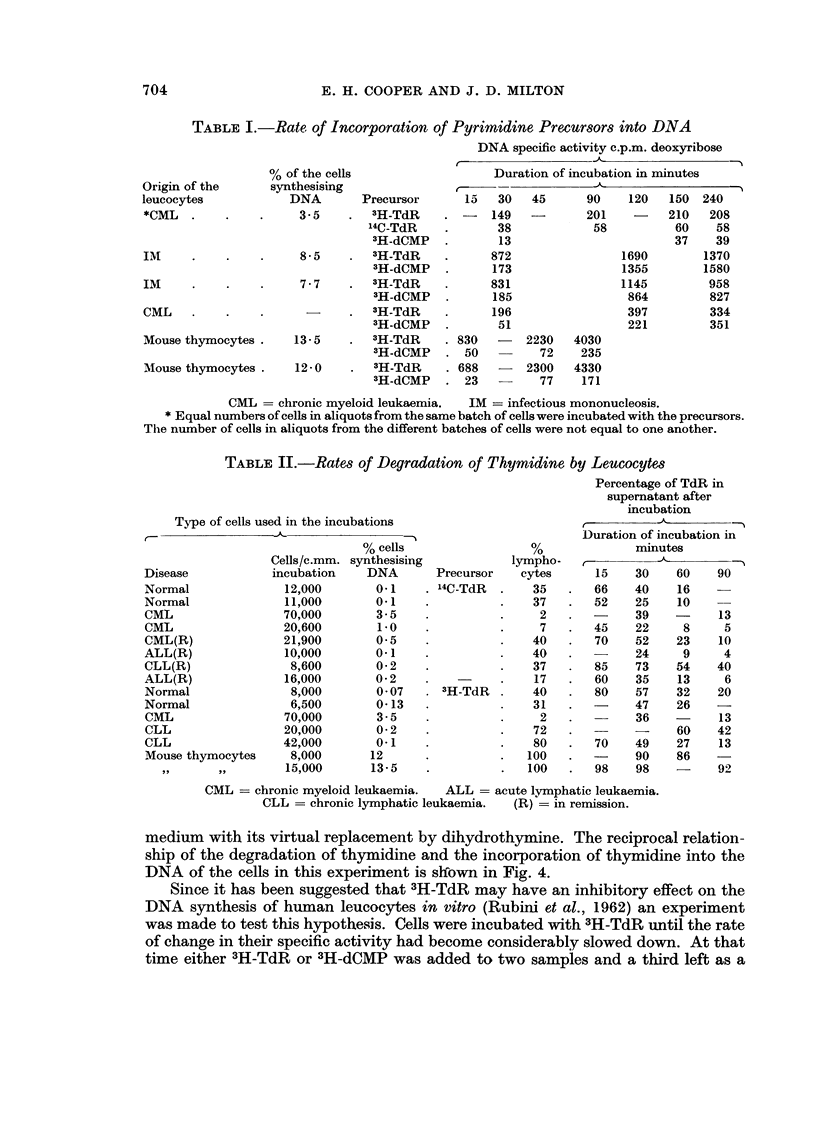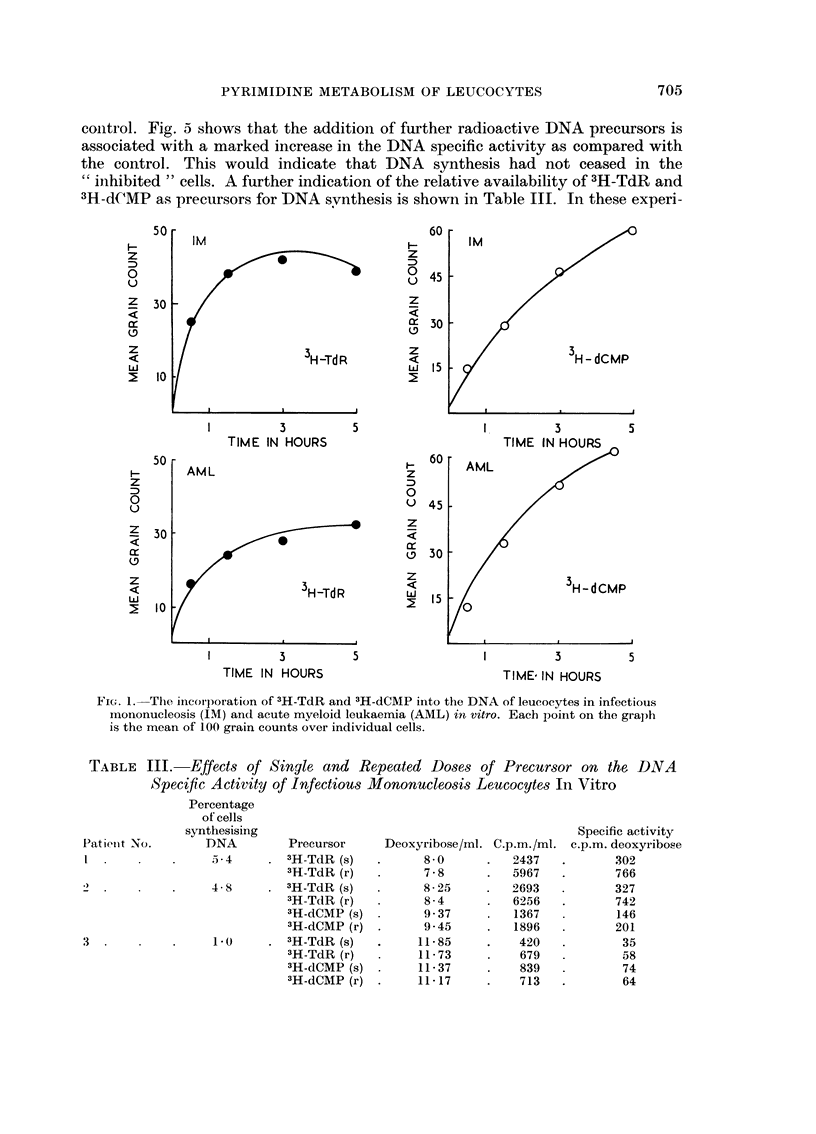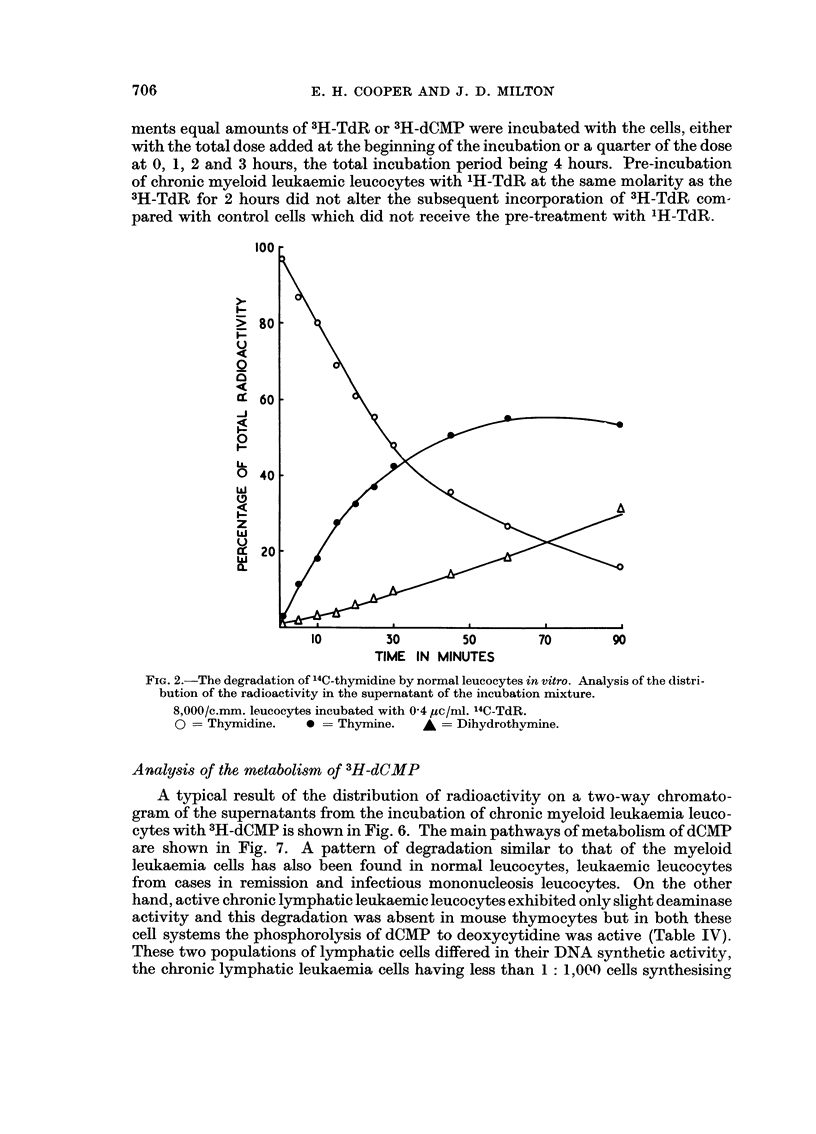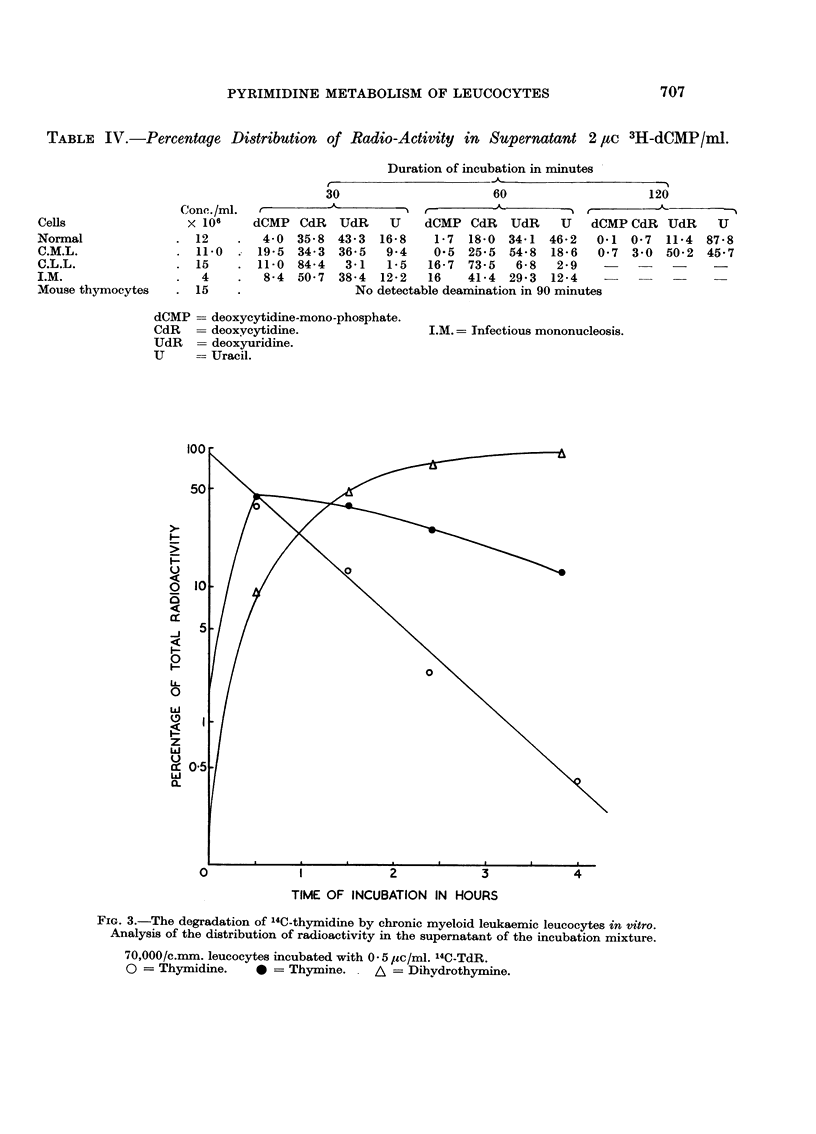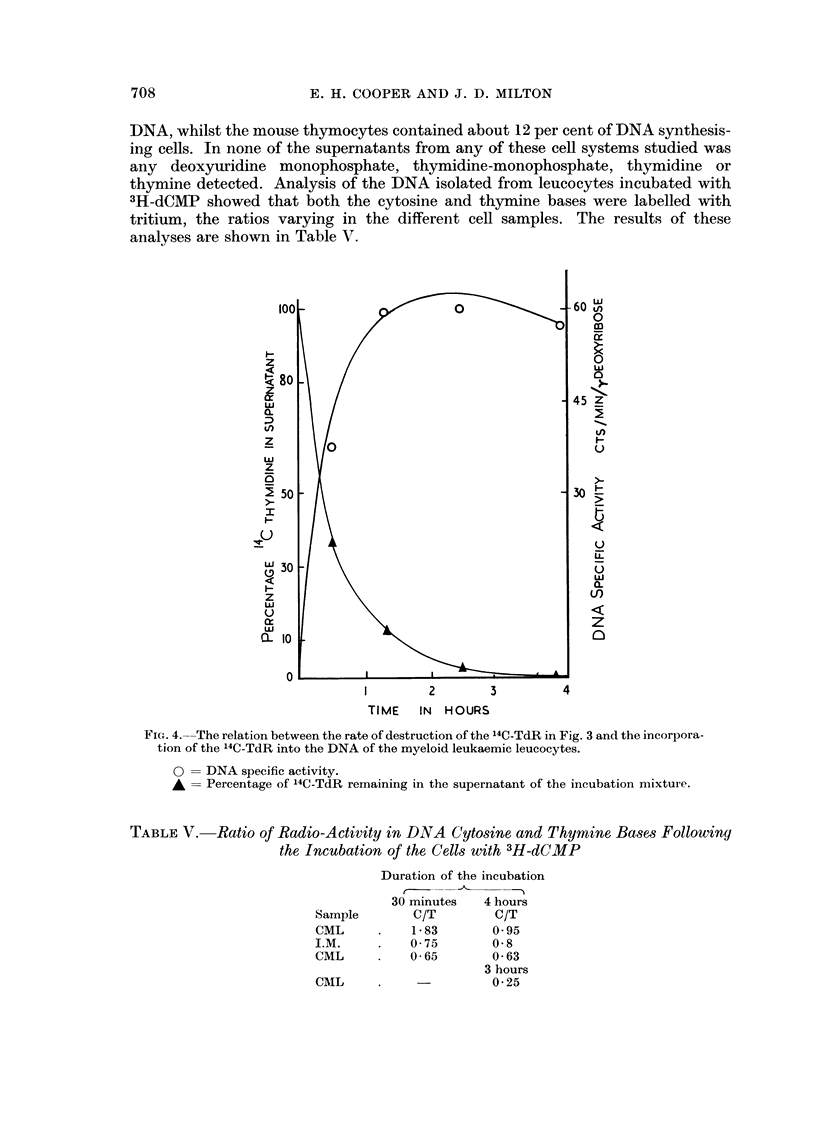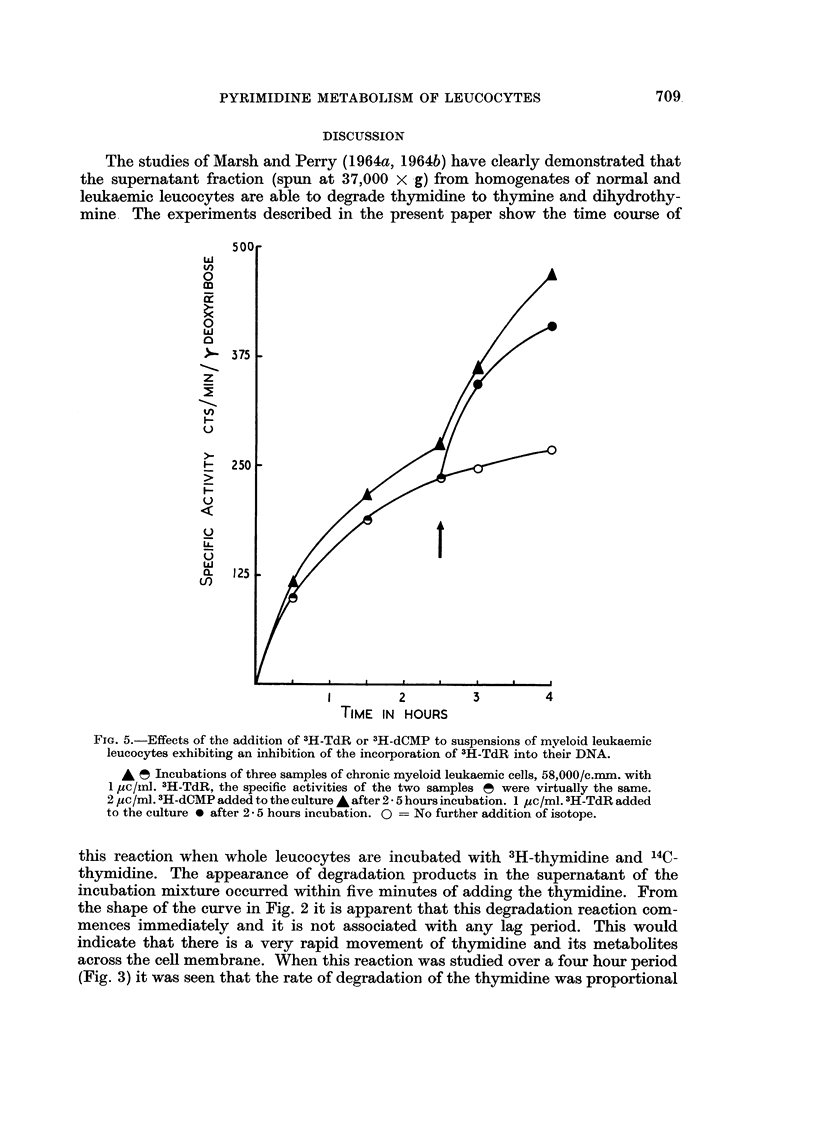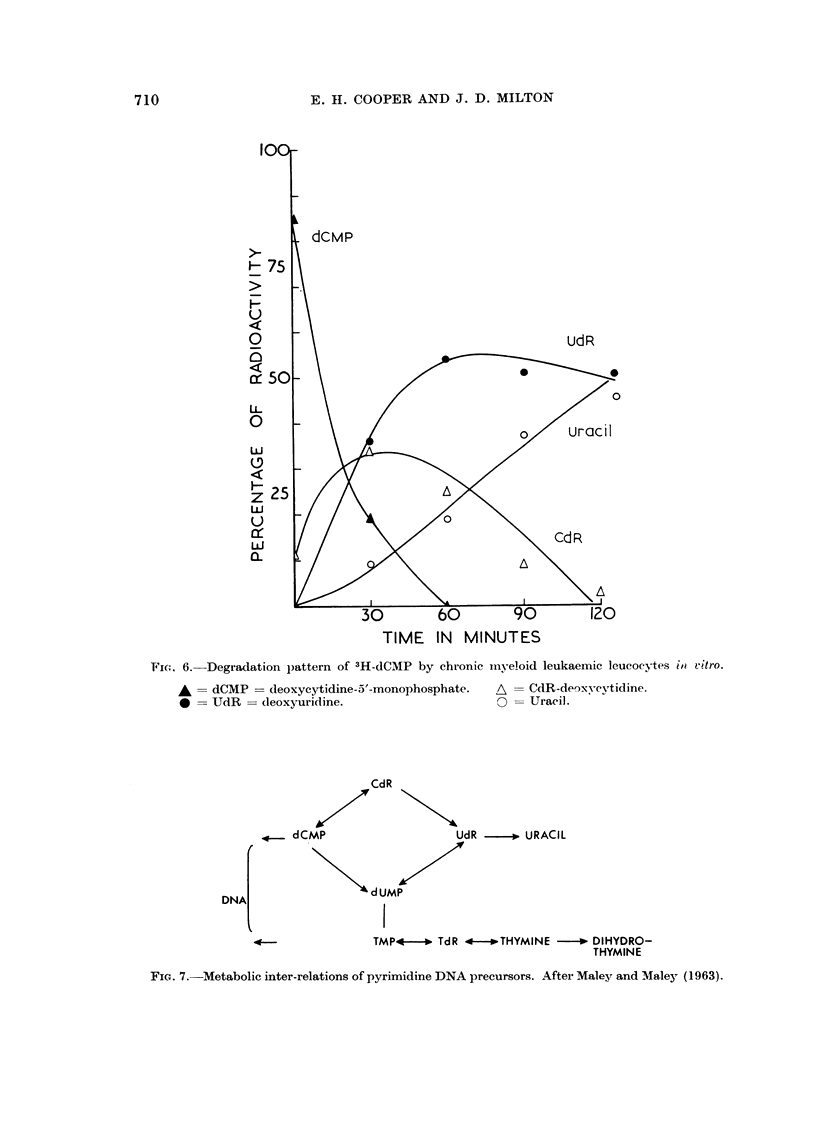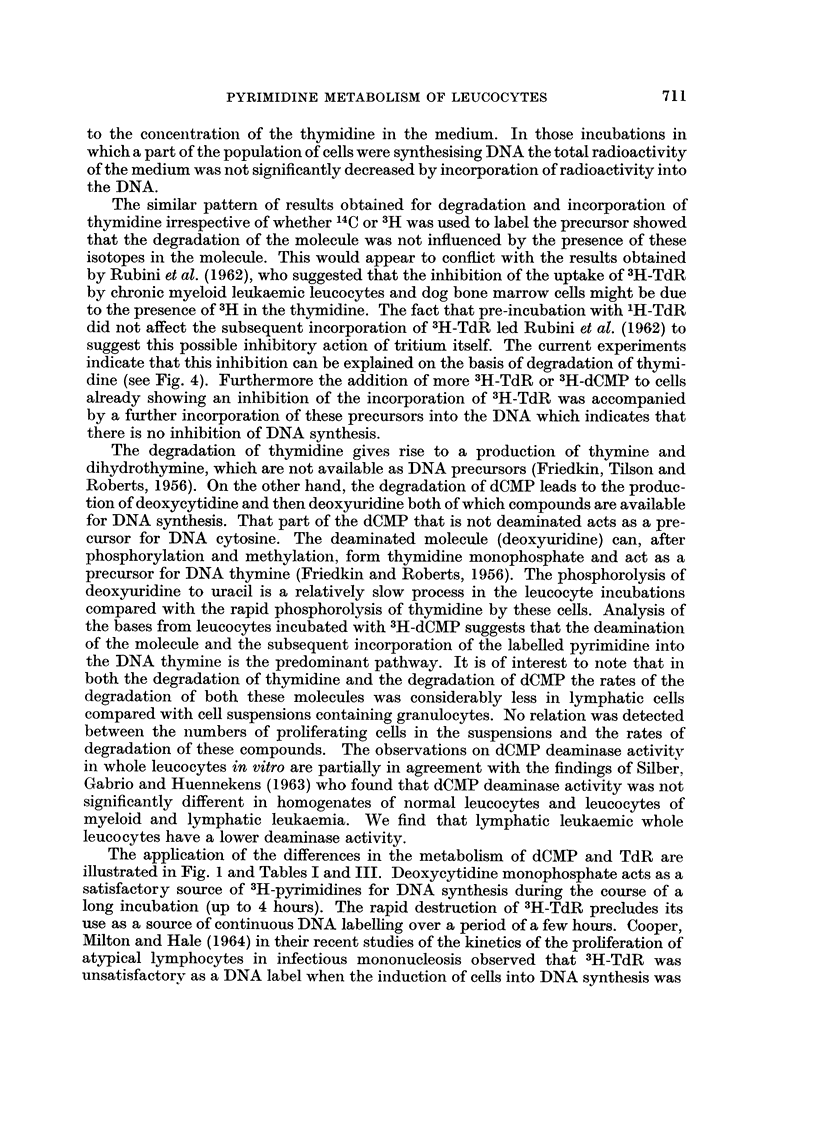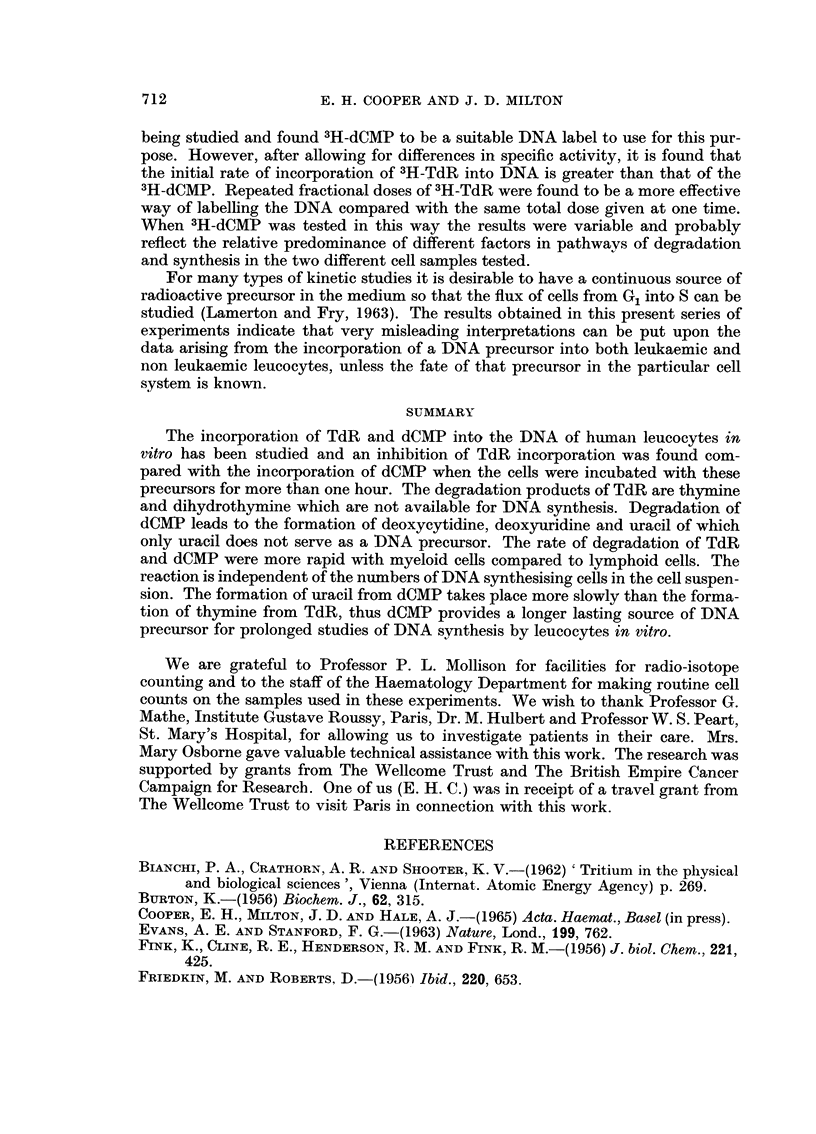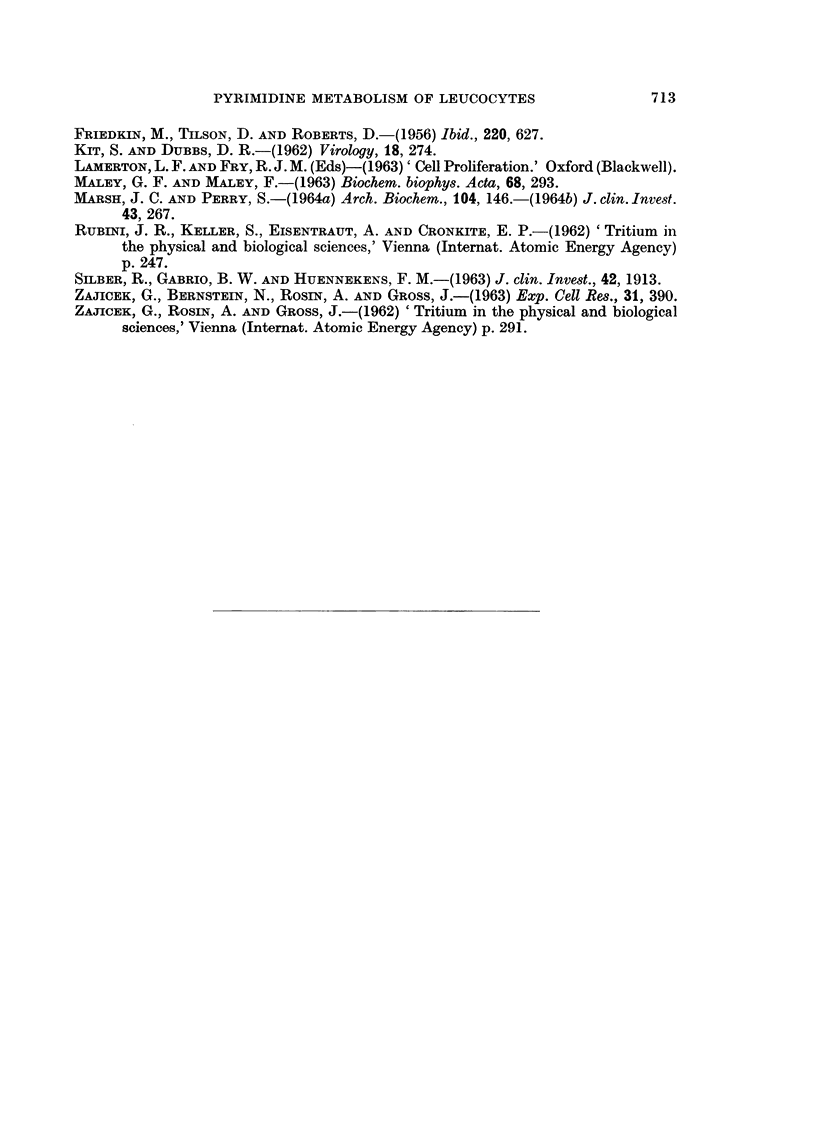# The Incorporation and Degradation of Pyrimidine DNA Precursors by Human Leucocytes

**DOI:** 10.1038/bjc.1964.81

**Published:** 1964-12

**Authors:** E. H. Cooper, J. D. Milton


### THE INCORPORATION AND DEGRADATION OF PYRIMIDINE DNA PRECURSORS BY HUMAN LEUCOCYTES

E. H. COOPER* AND J. D. MILTON

*From the Medical Unit, St. Mary's Hospital Medical School, London, W.2*

Received for publication September 24, 1964

TRITIATED thymidine is well recognised to be a highly specific precursor for DNA synthesis in mammalian cells. During the past few years there have been a number of reports of an inhibition of incorporation of $^{3}$H-thymidine ($^{3}$H-TdR) into the DNA of myeloid leukaemic leucocytes, bone marrow cells and ascites tumour cells *in vitro*, when these cells had been incubated with this precursor for an hour or more (Bianchi, Crathorn and Shooter, 1962 ; Rubini, Keller, Eisentraut and Cronkite, 1962 ; Zajicek, Rosin and Gross, 1962). At this time the reasons for this inhibition were obscure. Zajicek, Bernstein, Rosin and Gross (1963) reported the formation of radioactive thymine in the supernatant of a suspension of ascites cells incubated for one hour with $^{3}$H-TdR. More recently Marsh and Perry (1964*a*, 1964*b*) have demonstrated that the supernatant fraction of homogenates of human leucocytes can degrade $^{3}$H-thymidine to thymine and dihydrothymine.

We have examined the relationship of the incorporation of $^{3}$H-TdR and $^{14}$C-TdR into the DNA of leucocytes to the degradation of this compound by the cells. A study has also been made of the metabolism by leucocytes of an alternative DNA precursor tritiated deoxycytidine-5′-monophosphate ($^{3}$H-dCMP). This investigation was made to see whether $^{3}$H-dCMP is a more useful DNA precursor than $^{3}$H-TdR when prolonged incubations in the presence of the radioactive label are required, such as in the analysis of the kinetic pattern of the proliferation of leukaemic cells *in vitro*.

### MATERIALS AND METHODS

*Leucocyte suspensions.*—Peripheral venous blood was collected into heparinised bottles, which were stood for approximately 30 minutes at 37° C. after which time the red blood cells had sedimented out leaving a leucocyte rich plasma which was removed. The experiments were carried out with the cells suspended either in undiluted plasma or in plasma diluted 1/1 v/v with tissue culture medium TC 199 (Glaxo, Greenford, Middlesex). Leucocytes were obtained from patients with infectious mononucleosis, myeloid or lymphatic leukaemia and from normal subjects. The mouse thymocytes were prepared from $A_2G$ mice and suspended in Hanks' solution containing 20 per cent mouse serum.

*Isotopes.*—Tritiated thymidine ($^{3}$H-TdR) specific activity 2–3 C/mM and 2-$^{14}$C-thymidine ($^{14}$C-TdR) specific activity 30·4 mc/mM were obtained from the Radiochemical Centre, Amersham, Bucks. Tritiated deoxycytidine-5′-monophosphate ($^{3}$H-dCMP) specific activity 1 C/mM and 2-$^{14}$C-deoxycytidine-5′-monophosphate ($^{14}$C-dCMP) specific activity 12 mc/mM were obtained from Schwarz Bioresearch Inc., Orangeburg, N.Y.

* Wellcome Trust Senior Research Fellow in Clinical Science.

*Incubation procedure.*—All incubations of leucocyte suspensions with the radiotive precursors were carried out at 37° C. In the experiments for investigation of uptake of radioactive precursor into the DNA the precursors were used at a concentration of 1–2 $\mu$c/ml. In the experiments concerned with the degradation of the precursors the concentrations varied and are given for each individual experiment. For the estimation of uptake of radioactivity into the DNA of individual cells by autoradiography samples were removed from the incubation mixtures at specific times, rapidly cooled, washed with ice cold Hanks' solution and then suspended in Hanks' solution at 37° diluted 3 : 1 v/v with distilled water for 7–10 minutes. Then the cells were fixed in methanol/acetic acid 3/1 v/v and dropped on to ice cold slides. For the investigation of the uptake of radioactivity into the DNA as measured by scintillation counting, samples from the incubation mixture were taken into 2 volumes of methanol and the DNA extracted from the precipitate formed. For degradation studies the samples from the incubation were again added to 2 volumes of methanol and the supernatant obtained from this procedure was analysed.

*Autoradiography.*—Usually the cell preparations were stained by the Feulgen method before autoradiographs were prepared, but in some cases the developed autoradiographs were stained through the film with McNeal tetrachrome stain at pH 6·5. Autoradiographs were prepared with Ilford K5 nuclear research emulsion exposed at 5° C. for times varying between 1–7 days. They were developed in Kodak 19B developer for 5 minutes, rinsed in tap water and fixed in Kodak acid fixing salt plus hardener for 7 minutes, all processes being carried out at 16° C.

*Isolation of DNA.*—DNA was isolated from the cells by extraction with 0·5 N perchloric acid using the method of Kit and Dubbs (1962). The quantity of DNA was estimated by the method of Burton (1956) and the radioactivity counted in a Packard Tricarb Liquid Scintillation Spectrometer. The counting vials contained 0·2 ml. perchloric acid solution containing the DNA, 2 ml. Hyamine (1 M in methanol), 1 ml. ethanol and 10 ml. scintillation fluid. The scintillation fluid consisted of 5 g. 2–5 diphenyl oxazole (PPO) and 0·3 g. 1·4-bis-2-(4-methyl-5 phenyl oxazolyl) benzene (dimethyl POPOP) dissolved in 1 litre of toluene.

*Analysis of the degradation products*

Thymidine and its degradation products were analysed by paper chromatography after the methods of Fink, Cline, Henderson and Fink (1956). The aqueous methanol supernatants from the incubation samples were chromatographed on Whatman No. 1 paper by ascending chromatography using as a solvent the upper layer from a mixture of ethyl acetate : water : formic acid 60 : 30 : 10. A beaker containing 20 ml. of the lower phase was placed in the bottom of the tank. Unlabelled thymidine and thymine (L. Light & Co.) and dihydrothymine (Sigma Chemical Coy.) were added to the paper and the positions of thymine and thymidine ascertained by absorption of ultra violet light at 254 m$\mu$ using a Hanovaria Chromatolite. Dihydrothymine was identified by spraying the paper first with 1 N NaoH and secondly, after 30 minutes, with Ehrlich's reagent (*p*-dimethyl aminobenzaldehyde in HCl and ethanol); this produced a yellow spot. The spots were then cut out of the chromatogram and counted in approx. 15 ml. of scintillation fluid.

In this solvent system dihydrothymine and its degradation product $\beta$ ureido isobutyric acid have almost identical Rf values. The dihydrothymine was further identified by ascending chromatography in two other solvent systems :—sec butanol : tert butanol : water 42·5 : 8·5 : 47·5 and butanol : water : 0·88 ammonia, 60 : 30 : 10 (Fink, Cline, Henderson and Fink, 1956). The degradation products of dCMP were partially identified by 1 dimensional ascending chromatography using as a solvent the upper phase from a mixture of ethyl acetate : water : formic acid 60 : 27·5 : 12·5. Complete identification was effected by 2 dimensional chromatography using the same solvent in the first direction and using for the second direction descending chromatography in tert butanol : water : conc. HCl, 11 : 3 : 1. By this method the following compounds could be separated :—dCMP, deoxyuridine-monophosphate (dUMP), thymidine monophosphate (TMP), deoxycytidine (CdR), deoxyuridine (UdR), uracil and thymine all being identified by their UV absorption. The spots were counted as for the thymidine degradation products. A further check on the distribution of the radioactivity in those samples containing $^{14}C$ was made by autoradiography of the chromatograms.

### RESULTS

*Purity of the radioactive precursors.*—As high specific activity tritiated compounds are liable to undergo radiolysis (Evans and Stanford, 1963) the composition of the precursors used in these experiments were analysed. $^{3}H$-TdR-analysis showed the distribution of radioactivity varied according to the sample of $^{3}H$-TdR used. The percentage distribution was within the following limits: $^{3}H$-TdR (83·3–58·1), $^{3}H$-thymine (12·1–23·7), $^{3}H$ dihydrothymine (4·6–18·2), for the total activity in these three compounds. 3H dCMP analysis showed ($^{3}H$ dCMP 84 %) $^{3}H$-deoxycytidine 3 %, and left on origin and not identified 12 %. $^{14}C$ thymidine was found to be stable having approximately 98 % of its radioactivity on the thymidine spot. In view of the radiolysis of the tritiated compounds zero time analyses were made on the degradation studies and the change in the composition of the distribution of radioactivity calculated in relation to the composition of the original precursor solution added to the incubation mixtures.

### *Incorporation of radioactive precursors into the DNA*

Typical examples of the incorporation of $^{3}H$-TdR compared with that of $^{3}H$-dCMP are shown in Fig. 1. These two experiments show the behaviour of proliferating leucocytes in a benign disease (IM infectious mononucleosis) and a malignant disease (AML acute myeloid leukaemia). Table I shows the time course of increase in the DNA specific activity in the cells incubated with $^{3}H$-TdR, $^{14}C$-TdR or $^{3}H$-dCMP. It will be noticed that the mouse thymocytes, which have a high frequency of DNA synthesising cells showed an almost linear incorporation of $^{3}H$-TdR which is in marked contrast with that found in the blood leucocytes. Analysis of the supernatants from the various incubations with $^{3}H$-TdR and $^{14}C$TdR shows that there is a variable disappearance of the TdR from the medium (Table II). It will be noticed that in the mouse thymocytes, the same incubations as in Table I, the rate of disappearance of the thymidine was very slow. Fig. 2 confirms the observations of Marsh and Perry (1964*b*) that normal leucocytes degrade TdR *in vitro* and shows the rate of degradation of TdR and the corresponding rates of production of thymine and dihydrothymine. In Fig. 3 myeloid leukaemia cells have been incubated with $^{14}C$-TdR and the incubation prolonged to 4 hours. Under these conditions there is an almost complete removal of the thymidine from the

TABLE I.—*Rate of Incorporation of Pyrimidine Precursors into DNA*

| Origin of the leucocytes | % of the cells synthesising DNA | Precursor | DNA specific activity c.p.m. deoxyribose — Duration of incubation in minutes: 15 | 30 | 45 | 90 | 120 | 150 | 240 |
|---|---|---|---|---|---|---|---|---|---|
| *CML | 3·5 | $^{3}H$-TdR | — | 149 | — | 201 | — | 210 | 208 |
| | | $^{14}C$-TdR | | 38 | | 58 | | 60 | 58 |
| | | $^{3}H$-dCMP | | 13 | | | | 37 | 39 |
| IM | 8·5 | $^{3}H$-TdR | | 872 | | | 1690 | | 1370 |
| | | $^{3}H$-dCMP | | 173 | | | 1355 | | 1580 |
| IM | 7·7 | $^{3}H$-TdR | | 831 | | | 1145 | | 958 |
| | | $^{3}H$-dCMP | | 185 | | | 864 | | 827 |
| CML | — | $^{3}H$-TdR | | 196 | | | 397 | | 334 |
| | | $^{3}H$-dCMP | | 51 | | | 221 | | 351 |
| Mouse thymocytes | 13·5 | $^{3}H$-TdR | 830 | — | 2230 | 4030 | | | |
| | | $^{3}H$-dCMP | 50 | — | 72 | 235 | | | |
| Mouse thymocytes | 12·0 | $^{3}H$-TdR | 688 | — | 2300 | 4330 | | | |
| | | $^{3}H$-dCMP | 23 | — | 77 | 171 | | | |

CML = chronic myeloid leukaemia. IM = infectious mononucleosis.
* Equal numbers of cells in aliquots from the same batch of cells were incubated with the precursors. The number of cells in aliquots from the different batches of cells were not equal to one another.

TABLE II.—*Rates of Degradation of Thymidine by Leucocytes*

| Type of cells used in the incubations: Disease | Cells/c.mm. incubation | % cells synthesising DNA | Precursor | % lympho-cytes | Percentage of TdR in supernatant after incubation — Duration of incubation in minutes: 15 | 30 | 60 | 90 |
|---|---|---|---|---|---|---|---|---|
| Normal | 12,000 | 0·1 | $^{14}C$-TdR | 35 | 66 | 40 | 16 | — |
| Normal | 11,000 | 0·1 | | 37 | 52 | 25 | 10 | — |
| CML | 70,000 | 3·5 | | 2 | — | 39 | — | 13 |
| CML | 20,600 | 1·0 | | 7 | 45 | 22 | 8 | 5 |
| CML(R) | 21,900 | 0·5 | | 40 | 70 | 52 | 23 | 10 |
| ALL(R) | 10,000 | 0·1 | | 40 | — | 24 | 9 | 4 |
| CLL(R) | 8,600 | 0·2 | | 37 | 85 | 73 | 54 | 40 |
| ALL(R) | 16,000 | 0·2 | — | 17 | 60 | 35 | 13 | 6 |
| Normal | 8,000 | 0·07 | $^{3}H$-TdR | 40 | 80 | 57 | 32 | 20 |
| Normal | 6,500 | 0·13 | | 31 | — | 47 | 26 | — |
| CML | 70,000 | 3·5 | | 2 | — | 36 | — | 13 |
| CLL | 20,000 | 0·2 | | 72 | — | — | 60 | 42 |
| CLL | 42,000 | 0·1 | | 80 | 70 | 49 | 27 | 13 |
| Mouse thymocytes | 8,000 | 12 | | 100 | — | 90 | 86 | — |
| „ „ | 15,000 | 13·5 | | 100 | 98 | 98 | — | 92 |

CML = chronic myeloid leukaemia. ALL = acute lymphatic leukaemia.
CLL = chronic lymphatic leukaemia. (R) = in remission.

medium with its virtual replacement by dihydrothymine. The reciprocal relationship of the degradation of thymidine and the incorporation of thymidine into the DNA of the cells in this experiment is shown in Fig. 4.

Since it has been suggested that $^{3}H$-TdR may have an inhibitory effect on the DNA synthesis of human leucocytes *in vitro* (Rubini *et al.*, 1962) an experiment was made to test this hypothesis. Cells were incubated with $^{3}H$-TdR until the rate of change in their specific activity had become considerably slowed down. At that time either $^{3}H$-TdR or $^{3}H$-dCMP was added to two samples and a third left as a control. Fig. 5 shows that the addition of further radioactive DNA precursors is associated with a marked increase in the DNA specific activity as compared with the control. This would indicate that DNA synthesis had not ceased in the "inhibited" cells. A further indication of the relative availability of $^3$H-TdR and $^3$H-dCMP as precursors for DNA synthesis is shown in Table III. In these experi-

FIG. 1.—The incorporation of $^3$H-TdR and $^3$H-dCMP into the DNA of leucocytes in infectious mononucleosis (IM) and acute myeloid leukaemia (AML) *in vitro*. Each point on the graph is the mean of 100 grain counts over individual cells.

TABLE III.—*Effects of Single and Repeated Doses of Precursor on the DNA Specific Activity of Infectious Mononucleosis Leucocytes* In Vitro

| Patient No. | Percentage of cells synthesising DNA | Precursor | Deoxyribose/ml. | C.p.m./ml. | Specific activity c.p.m. deoxyribose |
|---|---|---|---|---|---|
| 1 | 5·4 | $^3$H-TdR (s) | 8·0 | 2437 | 302 |
| | | $^3$H-TdR (r) | 7·8 | 5967 | 766 |
| 2 | 4·8 | $^3$H-TdR (s) | 8·25 | 2693 | 327 |
| | | $^3$H-TdR (r) | 8·4 | 6256 | 742 |
| | | $^3$H-dCMP (s) | 9·37 | 1367 | 146 |
| | | $^3$H-dCMP (r) | 9·45 | 1896 | 201 |
| 3 | 1·0 | $^3$H-TdR (s) | 11·85 | 420 | 35 |
| | | $^3$H-TdR (r) | 11·73 | 679 | 58 |
| | | $^3$H-dCMP (s) | 11·37 | 839 | 74 |
| | | $^3$H-dCMP (r) | 11·17 | 713 | 64 |

ments equal amounts of $^{3}$H-TdR or $^{3}$H-dCMP were incubated with the cells, either with the total dose added at the beginning of the incubation or a quarter of the dose at 0, 1, 2 and 3 hours, the total incubation period being 4 hours. Pre-incubation of chronic myeloid leukaemic leucocytes with $^{1}$H-TdR at the same molarity as the $^{3}$H-TdR for 2 hours did not alter the subsequent incorporation of $^{3}$H-TdR compared with control cells which did not receive the pre-treatment with $^{1}$H-TdR.

FIG. 2.—The degradation of $^{14}$C-thymidine by normal leucocytes *in vitro*. Analysis of the distribution of the radioactivity in the supernatant of the incubation mixture.

8,000/c.mm. leucocytes incubated with 0·4 $\mu$c/ml. $^{14}$C-TdR.

○ = Thymidine. ● = Thymine. ▲ = Dihydrothymine.

*Analysis of the metabolism of $^{3}$H-dCMP*

A typical result of the distribution of radioactivity on a two-way chromatogram of the supernatants from the incubation of chronic myeloid leukaemia leucocytes with $^{3}$H-dCMP is shown in Fig. 6. The main pathways of metabolism of dCMP are shown in Fig. 7. A pattern of degradation similar to that of the myeloid leukaemia cells has also been found in normal leucocytes, leukaemic leucocytes from cases in remission and infectious mononucleosis leucocytes. On the other hand, active chronic lymphatic leukaemic leucocytes exhibited only slight deaminase activity and this degradation was absent in mouse thymocytes but in both these cell systems the phosphorolysis of dCMP to deoxycytidine was active (Table IV). These two populations of lymphatic cells differed in their DNA synthetic activity, the chronic lymphatic leukaemia cells having less than 1 : 1,000 cells synthesising

TABLE IV.—*Percentage Distribution of Radio-Activity in Supernatant* 2 $\mu$c $^3$H-dCMP/ml.

| | | Duration of incubation in minutes | | | | | | | | | | | |
|---|---|---|---|---|---|---|---|---|---|---|---|---|---|
| | | 30 | | | | 60 | | | | 120 | | | |
| Cells | Conc./ml. × $10^6$ | dCMP | CdR | UdR | U | dCMP | CdR | UdR | U | dCMP | CdR | UdR | U |
| Normal | 12 | 4·0 | 35·8 | 43·3 | 16·8 | 1·7 | 18·0 | 34·1 | 46·2 | 0·1 | 0·7 | 11·4 | 87·8 |
| C.M.L. | 11·0 | 19·5 | 34·3 | 36·5 | 9·4 | 0·5 | 25·5 | 54·8 | 18·6 | 0·7 | 3·0 | 50·2 | 45·7 |
| C.L.L. | 15 | 11·0 | 84·4 | 3·1 | 1·5 | 16·7 | 73·5 | 6·8 | 2·9 | — | — | — | — |
| I.M. | 4 | 8·4 | 50·7 | 38·4 | 12·2 | 16 | 41·4 | 29·3 | 12·4 | — | — | — | — |
| Mouse thymocytes | 15 | No detectable deamination in 90 minutes | | | | | | | | | | | |

dCMP = deoxycytidine-mono-phosphate.

CdR = deoxycytidine.

UdR = deoxyuridine.

U = Uracil.

I.M. = Infectious mononucleosis.

FIG. 3.—The degradation of $^{14}$C-thymidine by chronic myeloid leukaemic leucocytes *in vitro*. Analysis of the distribution of radioactivity in the supernatant of the incubation mixture.

70,000/c.mm. leucocytes incubated with 0·5 $\mu$c/ml. $^{14}$C-TdR.

○ = Thymidine. ● = Thymine. △ = Dihydrothymine.

DNA, whilst the mouse thymocytes contained about 12 per cent of DNA synthesising cells. In none of the supernatants from any of these cell systems studied was any deoxyuridine monophosphate, thymidine-monophosphate, thymidine or thymine detected. Analysis of the DNA isolated from leucocytes incubated with $^{3}$H-dCMP showed that both the cytosine and thymine bases were labelled with tritium, the ratios varying in the different cell samples. The results of these analyses are shown in Table V.

FIG. 4.—The relation between the rate of destruction of the $^{14}$C-TdR in Fig. 3 and the incorporation of the $^{14}$C-TdR into the DNA of the myeloid leukaemic leucocytes.

○ = DNA specific activity.
▲ = Percentage of $^{14}$C-TdR remaining in the supernatant of the incubation mixture.

TABLE V.—*Ratio of Radio-Activity in DNA Cytosine and Thymine Bases Following the Incubation of the Cells with $^{3}$H-dCMP*

| | Duration of the incubation | |
|---|---|---|
| | 30 minutes | 4 hours |
| Sample | C/T | C/T |
| CML . | 1·83 | 0·95 |
| I.M. . | 0·75 | 0·8 |
| CML . | 0·65 | 0·63 |
| | | 3 hours |
| CML . | — | 0·25 |

### DISCUSSION

The studies of Marsh and Perry (1964*a*, 1964*b*) have clearly demonstrated that the supernatant fraction (spun at 37,000 × g) from homogenates of normal and leukaemic leucocytes are able to degrade thymidine to thymine and dihydrothymine. The experiments described in the present paper show the time course of

FIG. 5.—Effects of the addition of $^{3}$H-TdR or $^{3}$H-dCMP to suspensions of myeloid leukaemic leucocytes exhibiting an inhibition of the incorporation of $^{3}$H-TdR into their DNA.

▲ ◓ Incubations of three samples of chronic myeloid leukaemic cells, 58,000/c.mm. with 1 $\mu$c/ml. $^{3}$H-TdR, the specific activities of the two samples ◓ were virtually the same. 2 $\mu$c/ml. $^{3}$H-dCMP added to the culture ▲ after 2·5 hours incubation. 1 $\mu$c/ml. $^{3}$H-TdR added to the culture ● after 2·5 hours incubation. ○ = No further addition of isotope.

this reaction when whole leucocytes are incubated with $^{3}$H-thymidine and $^{14}$C-thymidine. The appearance of degradation products in the supernatant of the incubation mixture occurred within five minutes of adding the thymidine. From the shape of the curve in Fig. 2 it is apparent that this degradation reaction commences immediately and it is not associated with any lag period. This would indicate that there is a very rapid movement of thymidine and its metabolites across the cell membrane. When this reaction was studied over a four hour period (Fig. 3) it was seen that the rate of degradation of the thymidine was proportional

FIG. 6.—Degradation pattern of $^{3}$H-dCMP by chronic myeloid leukaemic leucocytes *in vitro*.

▲ = dCMP = deoxycytidine-5′-monophosphate. △ = CdR-deoxycytidine.

● = UdR = deoxyuridine. ○ = Uracil.

FIG. 7.—Metabolic inter-relations of pyrimidine DNA precursors. After Maley and Maley (1963).

to the concentration of the thymidine in the medium. In those incubations in which a part of the population of cells were synthesising DNA the total radioactivity of the medium was not significantly decreased by incorporation of radioactivity into the DNA.

The similar pattern of results obtained for degradation and incorporation of thymidine irrespective of whether $^{14}C$ or $^{3}H$ was used to label the precursor showed that the degradation of the molecule was not influenced by the presence of these isotopes in the molecule. This would appear to conflict with the results obtained by Rubini *et al.* (1962), who suggested that the inhibition of the uptake of $^{3}H$-TdR by chronic myeloid leukaemic leucocytes and dog bone marrow cells might be due to the presence of $^{3}H$ in the thymidine. The fact that pre-incubation with $^{1}H$-TdR did not affect the subsequent incorporation of $^{3}H$-TdR led Rubini *et al.* (1962) to suggest this possible inhibitory action of tritium itself. The current experiments indicate that this inhibition can be explained on the basis of degradation of thymidine (see Fig. 4). Furthermore the addition of more $^{3}H$-TdR or $^{3}H$-dCMP to cells already showing an inhibition of the incorporation of $^{3}H$-TdR was accompanied by a further incorporation of these precursors into the DNA which indicates that there is no inhibition of DNA synthesis.

The degradation of thymidine gives rise to a production of thymine and dihydrothymine, which are not available as DNA precursors (Friedkin, Tilson and Roberts, 1956). On the other hand, the degradation of dCMP leads to the production of deoxycytidine and then deoxyuridine both of which compounds are available for DNA synthesis. That part of the dCMP that is not deaminated acts as a precursor for DNA cytosine. The deaminated molecule (deoxyuridine) can, after phosphorylation and methylation, form thymidine monophosphate and act as a precursor for DNA thymine (Friedkin and Roberts, 1956). The phosphorolysis of deoxyuridine to uracil is a relatively slow process in the leucocyte incubations compared with the rapid phosphorolysis of thymidine by these cells. Analysis of the bases from leucocytes incubated with $^{3}H$-dCMP suggests that the deamination of the molecule and the subsequent incorporation of the labelled pyrimidine into the DNA thymine is the predominant pathway. It is of interest to note that in both the degradation of thymidine and the degradation of dCMP the rates of the degradation of both these molecules was considerably less in lymphatic cells compared with cell suspensions containing granulocytes. No relation was detected between the numbers of proliferating cells in the suspensions and the rates of degradation of these compounds. The observations on dCMP deaminase activity in whole leucocytes *in vitro* are partially in agreement with the findings of Silber, Gabrio and Huennekens (1963) who found that dCMP deaminase activity was not significantly different in homogenates of normal leucocytes and leucocytes of myeloid and lymphatic leukaemia. We find that lymphatic leukaemic whole leucocytes have a lower deaminase activity.

The application of the differences in the metabolism of dCMP and TdR are illustrated in Fig. 1 and Tables I and III. Deoxycytidine monophosphate acts as a satisfactory source of $^{3}H$-pyrimidines for DNA synthesis during the course of a long incubation (up to 4 hours). The rapid destruction of $^{3}H$-TdR precludes its use as a source of continuous DNA labelling over a period of a few hours. Cooper, Milton and Hale (1964) in their recent studies of the kinetics of the proliferation of atypical lymphocytes in infectious mononucleosis observed that $^{3}H$-TdR was unsatisfactory as a DNA label when the induction of cells into DNA synthesis was being studied and found $^3$H-dCMP to be a suitable DNA label to use for this purpose. However, after allowing for differences in specific activity, it is found that the initial rate of incorporation of $^3$H-TdR into DNA is greater than that of the $^3$H-dCMP. Repeated fractional doses of $^3$H-TdR were found to be a more effective way of labelling the DNA compared with the same total dose given at one time. When $^3$H-dCMP was tested in this way the results were variable and probably reflect the relative predominance of different factors in pathways of degradation and synthesis in the two different cell samples tested.

For many types of kinetic studies it is desirable to have a continuous source of radioactive precursor in the medium so that the flux of cells from $G_1$ into S can be studied (Lamerton and Fry, 1963). The results obtained in this present series of experiments indicate that very misleading interpretations can be put upon the data arising from the incorporation of a DNA precursor into both leukaemic and non leukaemic leucocytes, unless the fate of that precursor in the particular cell system is known.

### SUMMARY

The incorporation of TdR and dCMP into the DNA of human leucocytes *in vitro* has been studied and an inhibition of TdR incorporation was found compared with the incorporation of dCMP when the cells were incubated with these precursors for more than one hour. The degradation products of TdR are thymine and dihydrothymine which are not available for DNA synthesis. Degradation of dCMP leads to the formation of deoxycytidine, deoxyuridine and uracil of which only uracil does not serve as a DNA precursor. The rate of degradation of TdR and dCMP were more rapid with myeloid cells compared to lymphoid cells. The reaction is independent of the numbers of DNA synthesising cells in the cell suspension. The formation of uracil from dCMP takes place more slowly than the formation of thymine from TdR, thus dCMP provides a longer lasting source of DNA precursor for prolonged studies of DNA synthesis by leucocytes *in vitro*.

We are grateful to Professor P. L. Mollison for facilities for radio-isotope counting and to the staff of the Haematology Department for making routine cell counts on the samples used in these experiments. We wish to thank Professor G. Mathe, Institute Gustave Roussy, Paris, Dr. M. Hulbert and Professor W. S. Peart, St. Mary's Hospital, for allowing us to investigate patients in their care. Mrs. Mary Osborne gave valuable technical assistance with this work. The research was supported by grants from The Wellcome Trust and The British Empire Cancer Campaign for Research. One of us (E. H. C.) was in receipt of a travel grant from The Wellcome Trust to visit Paris in connection with this work.

### REFERENCES

Bianchi, P. A., Crathorn, A. R. and Shooter, K. V.—(1962) 'Tritium in the physical and biological sciences', Vienna (Internat. Atomic Energy Agency) p. 269.
Burton, K.—(1956) *Biochem. J.*, **62**, 315.
Cooper, E. H., Milton, J. D. and Hale, A. J.—(1965) *Acta. Haemat., Basel* (in press).
Evans, A. E. and Stanford, F. G.—(1963) *Nature*, Lond., **199**, 762.
Fink, K., Cline, R. E., Henderson, R. M. and Fink, R. M.—(1956) *J. biol. Chem.*, **221**, 425.
Friedkin, M. and Roberts, D.—(1956) *Ibid.*, **220**, 653.

FRIEDKIN, M., TILSON, D. AND ROBERTS, D.—(1956) *Ibid.*, **220**, 627.
KIT, S. AND DUBBS, D. R.—(1962) *Virology*, **18**, 274.
LAMERTON, L. F. AND FRY, R. J. M. (Eds)—(1963) 'Cell Proliferation.' Oxford (Blackwell).
MALEY, G. F. AND MALEY, F.—(1963) *Biochem. biophys. Acta*, **68**, 293.
MARSH, J. C. AND PERRY, S.—(1964*a*) *Arch. Biochem.*, **104**, 146.—(1964*b*) *J. clin. Invest.* **43**, 267.
RUBINI, J. R., KELLER, S., EISENTRAUT, A. AND CRONKITE, E. P.—(1962) 'Tritium in the physical and biological sciences,' Vienna (Internat. Atomic Energy Agency) p. 247.
SILBER, R., GABRIO, B. W. AND HUENNEKENS, F. M.—(1963) *J. clin. Invest.*, **42**, 1913.
ZAJICEK, G., BERNSTEIN, N., ROSIN, A. AND GROSS, J.—(1963) *Exp. Cell Res.*, **31**, 390.
ZAJICEK, G., ROSIN, A. AND GROSS, J.—(1962) 'Tritium in the physical and biological sciences,' Vienna (Internat. Atomic Energy Agency) p. 291.